# Semantic Data Mining in Ubiquitous Sensing: A Survey

**DOI:** 10.3390/s21134322

**Published:** 2021-06-24

**Authors:** Grzegorz J. Nalepa, Szymon Bobek, Krzysztof Kutt, Martin Atzmueller

**Affiliations:** 1 Institute of Applied Computer Science and Jagiellonian Human-Centered Artificial Intelligence Laboratory (JAHCAI), ul. Prof. Stanislawa Lojasiewicza 11, Jagiellonian University, 30-348 Krakow, Poland; szymon.bobek@uj.edu.pl (S.B.); krzysztof.kutt@uj.edu.pl (K.K.); 2 Department of Applied Computer Science, AGH University of Science and Technology, Al. Mickiewicza 30, 30-059 Krakow, Poland; 3 Semantic Information Systems Group, Osnabrück University, 49074 Osnabrück, Germany

**Keywords:** semantics, data mining, declarative methods, explainability, industrial sensors

## Abstract

Mining ubiquitous sensing data is important but also challenging, due to many factors, such as heterogeneous large-scale data that is often at various levels of abstraction. This also relates particularly to the important aspects of the explainability and interpretability of the applied models and their results, and thus ultimately to the outcome of the data mining process. With this, in general, the inclusion of domain knowledge leading towards semantic data mining approaches is an emerging and important research direction. This article aims to survey relevant works in these areas, focusing on semantic data mining approaches and methods, but also on selected applications of ubiquitous sensing in some of the most prominent current application areas. Here, we consider in particular: (1) environmental sensing; (2) ubiquitous sensing in industrial applications of artificial intelligence; and (3) social sensing relating to human interactions and the respective individual and collective behaviors. We discuss these in detail and conclude with a summary of this emerging field of research. In addition, we provide an outlook on future directions for semantic data mining in ubiquitous sensing contexts.

## 1. Introduction

With the advent of ubiquitous sensing driven by, for example, mobile computing, the Internet of Things and Industry 4.0, many novel research directions and interesting applications have emerged through the use of large-scale sensor data as well as advanced analysis and processing methods. While there is a multitude of such powerful processing and analytics methods in data mining (DM) and machine learning (ML), there are also specific challenges relating to the characteristics of the data in ubiquitous sensing. These include, for example, some of the common challenges of Big Data [1,2] such as volume, velocity and variety of the data. However, most importantly, aspects such as the interpretability and explainability of the applied data mining models and their results, respectively, motivate, require, or even enforce the application of domain/background knowledge to data mining and machine learning approaches.

In the context of ubiquitous sensing and sensor data processing, Big Data, for example, requires not only the proper selection and curation of potentially relevant data, but also the use of dimensionality reduction and feature construction and engineering. This also relates to making *Big Data* smart, that is, transforming it into *Smart Data* [3]. In particular, a number of important questions regarding the understandability and interpretability of machine learning models used in sensitive applications of DM and artificial intelligence (AI) (e.g.,  medicine) have recently been raised [4]. When applying the models for knowledge discovery and/or decision support, their transparency and explainability is often crucial, otherwise limiting acceptance and trust in their adoption in such sensitive contexts. Using background/domain knowledge enables semantic enrichment and ultimately semantic interpretation, leading to a knowledge-based analysis approach, which we call *semantic data mining*.

This article aims to survey relevant works in these areas, specifically focusing on different semantic data mining approaches and methods, but also on specific selected—current, prominent and emerging—application areas of ubiquitous sensing. Here, we consider in particular: (1) environmental sensing; (2) ubiquitous sensing in industrial applications of artificial intelligence; and, finally, (3) social sensing relating to human interactions—observing and capturing the respective individual and collective behaviors. We discuss these in detail and conclude with a broad outlook on future directions for semantic data mining approaches in ubiquitous sensing contexts.

There exist several comprehensive surveys on using semantic knowledge in data mining [5,6,7] or exploring the possibilities of combining data mining with background knowledge [8], and others that tackle the issue of moving from raw data to smart data [9,10,11,12]. However, they do not approach the subject holistically, instead focusing on particular aspects of semantic data mining or narrowing the view on how semantics can be defined and introduced to the data mining pipeline. We present a broader perspective on the issue and compare existing practical tools and frameworks that aid data scientists in building ubiquitous sensing systems.

Our contributions are summarized as follows:

1. We provide a comprehensive perspective on semantic data mining, including different methods and techniques from related areas to be captured under this common topic.
2. We discuss relevant applications in the context of ubiquitous sensing, exemplifying the specific implementation of semantic techniques in context.
3. We outline interesting future directions for the development and application of approaches and methods for semantic data mining in ubiquitous sensing.

The rest of the article is structured as follows: Section 2 provides an overview of semantic data mining, and in Section 3 we discuss specific application areas in ubiquitous sensing. Finally, Section 4 concludes with a summary and discusses interesting future challenges and perspectives for semantic data mining in ubiquitous sensing.

## 2. Overview of Semantic Data Mining Approaches

In the following, we structure the discussed relevant literature around the topics of data mining process models, semantic and declarative approaches for data mining, and the role of interpretability and explainability in DM.

### 2.1. Data Mining Process

The general goal of data mining is to uncover novel, interesting and ultimately understandable patterns [13]—that is, relating to valuable, useful and implicit knowledge. It is an iterative and often incremental process, such that a partial solution is often refined in order to arrive at the final one. There exist several approaches for data mining (see [14]), the most prominent of which is provided by the CRISP-DM process. It can be roughly divided into three sub-processes: domain focusing (understanding and data preparation); pattern modeling (the mining step); and model implementation (evaluation and deployment). CRISP-DM—consisting of six phases in total—is thus split into five iterative phases: Business Understanding (defining the goals of data mining); Data Understanding (making sure that data is applicable and clarifies semantics); Data Preparation (which usually needs about 80% of the total effort of the process for transforming and cleaning the data, including feature engineering, e.g.,  [15,16]); Modeling (the central phase: regularities and patterns are extracted from the data for constructing the data mining model); Evaluation (where the quality of the mined model needs to be assessed); and finally Deployment (where the model is applied, e.g., for pattern understanding, prediction, classification or clustering).

It is worth noting that CRISP-DM was proposed in the context of a long-time tradition of Knowledge Discovery from Databases (KDD) [13]. An overview [14] discusses the evolution of such approaches for KDD as well as for DM processes. Some of them include aspects that CRISP-DM omits, for example, Domain Knowledge Elicitation and Knowledge Post-processing. While a decade ago, the term “explanation” was rarely explicitly used, the explanatory aspect of the DM or KDD process was somewhat considered in the latter phases of related approaches, but still to a limited extent. Later, we will discuss how explanation is especially important in relation to our scope of much more complex DM approaches and processes today. This is, in particular, specifically relevant for semantic data mining in ubiquitous sensing due to the complex, heterogeneous, and typically uncertain and noisy, data.

Connected to this, some additional deficiencies were already identified a long time ago. First of all, it is a common opinion among DM experts that data understanding and preparation are typically the most costly and time-consuming (about 80%) phases in CRISP-DM, before even mentioning proper Business Understanding, for example, [15,16]. Moreover, the lack of feedback loops is emphasized [14].

To improve CRISP-DM, or to offer alternative solutions, more recently several new approaches have been proposed. The SAS Institute proposed its own SEMMA (Sample, Explore, Modify, Model and Assess) sequential approach for DM [17]; for a comparison with CRISP-DM see [18]. IBM proposed its own extension to the original CRISP-DM process, called ASUM-DM, to focus more on the operations side of implementing DM projects [19]. However, these two approaches remain sequential and do not consider the role of the domain knowledge, nor the explanative aspect. Most recently, the so-called *Model Development Process* was proposed [20]. It extends the ideas of Rational Unified Process, and partially considers the need to introduce explanations. However, it does not support the explicit elicitation of knowledge in any of the phases. In addition, there have been several proposals for including domain knowledge regarding the data mining process in general, for example, [21,22]. However, those mainly relate to the (declarative) specification of the process itself, or the “fine-tuning” of the process, but not to cross-links between the different steps of the process.

As such, in the next subsection we discuss a variety of approaches that aim to enhance data mining from a knowledge-oriented perspective.

### 2.2. Semantic, Knowledge-Based and Declarative Data Mining

Using background knowledge in DM has been proposed in the area of *semantic data mining*, where the knowledge is typically represented in a knowledge repository, such as an ontology or a knowledge base. The main aspect of semantic DM is the explicit integration of this knowledge into the DM and KDD modeling step, where the algorithms for data mining/modeling or post-processing make use of the formalized knowledge to improve the overall results. There has been growing interest in this issue (e.g., [23,24,25,26,27,28]) in various domains, for example in the medical domain [24,29,30,31] but also for industrial applications [26]. Here, [32] present a collaborative approach for specifying task-configurations of specific DM methods. Further examples include using ontologies in specific DM tasks (i.e.,  subgroup discovery and network analysis) [33,34].

However, in those approaches, domain knowledge is only used in a very specific setting, that is, modeling, so is not generalized to the whole DM process. The same observation holds for approaches that stress the importance of contextual knowledge for data mining, for example, [35,36], which applied context-aware approaches to the process.

Several toolkits allow for embedding *declarative knowledge* into the learning process. In [37], the authors proposed a neural network mechanism that allows for the representation of structured knowledge in the form of n-dimensional vectors. This is an approach equivalent to word2vec [38]. In [39], the usage of variational autoencoders for graph structure embedding was presented. Due to the variational nature of an encoder, it not only allows for embedding graphs, but also supports the generation of such. In particular, there have been various approaches in the fields of Semantic Web and Linked Open Data for DM, although their full potential is still to be unlocked [7]. Traditional DM processes still face major challenges in terms of massive data [40]. In addition, the application of data mining still faces serious challenges, one of which is reproducing already known knowledge. At the same time, DM systems typically make very little use of existing corporate knowledge [41]. Here, existing DM methodologies only provide general directions and directives, while users ultimately require explanations and recommendations on how to effectively perform the steps of the DM methodology. This is currently not provided or enabled by standard DM approaches, cf.  [42].

An idea for *declarative data analysis* is presented in [22], which specifically targets declarative problem formulation; however, they do not tackle a specific data mining process, nor augment the specific methods or connect between them explicitly. In the area of constraint programming there have been approaches (e.g.,  [43,44]) that re-frame a DM method using constraint-based programming. These approaches actually specify what the ML or DM task is about rather than utilizing contextual domain knowledge in a declarative way. So, this mainly relates to the interpretability of the specification of such approaches, not to the ultimate understandability/explainability of the process and/or its outcomes, since the proposed declarative systems mainly transform the declarative specifications in a kind of black-box manner [44].

### 2.3. Explainability and Interpretability in Data Mining

According to [45,46], the term ’explanation’ has been widely investigated in different disciplines. Explanations are in some sense always answers to questions, supporting humans in their decision-making [47]. In particular, explanations are a central component for advanced data mining approaches. This becomes especially relevant when considering complicated black-box models that provide recommendations and predictions in sensitive application contexts like medicine, Industry 4.0 and so forth. Here, nontransparent methods and models make it more difficult to spot errors and can thus lead to biased decisions. For example, this can be based on incorrect training data, or training data that is actually not suitable for application—for example, relating to its contained data quality. In general, nontransparent and non-explainable methods stretch the trust humans have (and should rightfully have) in the respective predictions. Then, the potential competitive advantage through better predictions for humans, for businesses, and for society as a whole comes at the cost of reduced explanatory power—which is specifically problematic for sensitive application areas like those for ubiquitous sensing. This is particularly important in the light of the European Union’s new General Data Protection Regulation and the “right to explanation” (providing users the right to obtain an explanation for any algorithmic decisions that were made about them), cf.  [48].

Recently, with these developments and more and more complex models, there has been growing interest in the development of so-called *eXplainable AI (XAI)* systems. One of the triggers was the NASA XAI Challenge [49]. From [50], XAI is described as “one that produces details or reasons to make its functioning clear or easy to understand.” That paper also outlines the differences between key concepts such as comprehensibility, interpretability, explainability, transparency, and the most important one: understandability (intelligibility).

As the challenges of XAI are mostly related to ML models and their use in the DM process, two main cases are considered: different levels of transparent ML models and post-hoc explainers for black-box ML models. Furthermore, we are interested in the hybrid approaches combining these two, for example, see [51]. For some authors, it is clear that the role of knowledge in the process of using proper ML models, and their use in the DM process, is paramount [52]. Furthermore, it is worth emphasizing that interpretability goes far beyond the model itself, and needs to be considered in the scope of the whole process of designing a system [53].

An overview of so-called interpretable ML techniques can be found in [4]. The interpretation of the results of ML explanation models, such as LIME [54], SHAP [55] or Anchor [56], highly depends on expert knowledge and domain knowledge. However, these frameworks do not provide any means for encoding such knowledge, relying purely on the *manual* examination and interpretation of their results. Many attempts have been made to aid domain experts or data scientists in the interpretation and incorporation of explanation results into the DM process. Most of them focus on the visual presentation of such results. This includes saliency maps for Deep Neural Networks [57], task specific visualisations [58] and more general frameworks [59], which are still narrowed to only one phase of the DM process and hardly use any domain knowledge to enhance explanations nor interpretability of the models. For some examples, in [60], the authors demonstrate how the combination of deep tensor and knowledge graph embedding methods can be used for generating explanations for a model in intrusion detection and genomic medicine. In [61], an approach aiming at predicting and explaining interactions between nodes in a knowledge graph is presented. In [62], an approach for exploiting knowledge graphs for the purpose of explanation is sketched. A medical ontology and temporal domain knowledge was successfully incorporated into the prediction model described in [63], for explaining decisions to the end-user.

In general, nontransparent methods and models make it more difficult to comprehend the decisions of the methods and models in general; also, it becomes very difficult to perform validation and to, for example, spot mistakes, since algorithmic methods can learn “bad habits” from the data. For example, if their training data contains misleading/wrongly classified examples, then it is highly likely that the resulting model incorporates specific biases induced by this training data. This can then simply lead to wrong conclusions and decisions due to, for example, incorrect or biased data capture, insufficient data preprocessing [64,65], and so forth. All these aspects are also particularly important and relevant in a ubiquitous sensing context, since we need to provide and ensure representative training and testing procedures, which can also be supported by the inclusion of semantic information and domain knowledge. In such cases, in general, the inability to provide an explanation as justification is a significant drawback of such methods, limiting acceptance and trust in their adoption in sensitive applications of DM—like those we discuss in the context of ubiquitous sensing. Therefore, interpretability and explainability are crucial for a successful DM process. This is enabled by developing computational methods—to “make sense” of complex information and knowledge processes—in a knowledge augmented DM approach. Here, the explanation has to be pushed through all the steps of the DM process.

There have been several attempts to provide methodological approaches for the evaluation and verification of given explanation results [66,67]. Among many qualitative approaches, there are also those that allow for quantitative evaluation. In [68], measures such as fidelity, consistency and stability were coined, which can be used for a numerical comparison of methods. In [69], the aforementioned measures were used to improve overall explanations. In [70], a measure that allows the capture of the stability or robustness of explanations was introduced. Context in terms of explanations is mostly considered in terms of the similarity of training instances within its vector space, not in the broader context of the domain. In [71], the authors exploit the context of features within a training instance to improve explanations generated with LIME. In [72], the context of an instance that is being explained is generated for the purpose of up-sampling and generating explanations. A more advanced approach was discussed in [73], where an interactive explanation architecture was presented that allows for interactive verification and ad-hoc personalization of the explanations.

The overview provided in this section emphasizes the role of knowledge and explanation in the DM process, and different approaches for introducing them. In the next section, we discuss selected illustrative examples of the applications of semantic data mining in ubiquitous sensing.

## 3. Applications in Ubiquitous Sensing

We distinguish different sensing areas and contexts that are mostly relevant to semantic DM approaches: environmental sensing, sensing in industrial artificial intelligence and social sensing. Here, we observe large scale and/or complex data, motivating the application of a semantic approach. Below, for data analysis, data mining and machine learning, we survey approaches and methods for the inclusion of domain knowledge in those specific contexts.

### 3.1. Environmental Sensing

Due to the fact that environmental and industrial (see Section 3.2) data originate from the sensors of multiple manufacturers, use different measurement methods and return data in a variety of measurement units, their use in DM requires appropriate semantization [12]. This is understood here as an appropriate formatting of data, enriching it with tags or labels and combining it with contextual knowledge to create a unified description that can be easily processed by automatic systems (e.g.,  DM tools) [10].

The most popular markups for sensory data semantization are [10] the following:

- Resource Description Framework (RDF) [74] and the Web Ontology Language (OWL) [75]—two Semantic Web standards developed by the World Wide Web Consortium (W3C). On their basis, many detailed models have been developed to describe a certain type of data or measurement context.
- Sensor Measurement Lists (SenML; in draft versions it was also called Sensor Markup Language) [76]—a standard aimed at small packets with simple sensor measurements that are easy to use in constrained networks, proposed by the Internet Engineering Task Force (IETF).
- Entity Notation (EN) [77]—another standard aimed at providing semantics for low-resource sensors. It provides the definition of short packets, which are transferred via communication links, and complete packets, derived from the short ones, useful for connection with ontologies.
- Observations and Measurements (O&M; https://www.ogc.org/standards/om; accessed on 13 April 2021) and Sensor Model Language (SensorML; https://www.ogc.org/standards/sensorml; accessed on 13 April 2021)—two complementary specifications proposed by the Open Geospatial Consortium (OGC) for observations and sensors description.

They differ not only in their expressivity, but also in their corresponding processing-related energy consumption [78]; this is important, as environmental and physiological sensing often takes place in real-time under resource-constraints in edge computing [79].

What these standards have in common is that they combine sensory data from various Internet of Things (IoT) devices into some kind of (knowledge) graph. The W3C stack of standards is the foundation of the Semantic Web. The SenML and EN notations do not constitute the semantic graph itself, but they can be easily translated into RDF (see [80] and [77], respectively). The O&M and SensorML standards are part of a broader set of services and languages developed by OGC for the Semantic Sensor Web [81]. They can also be integrated with the Semantic Web stack of technologies [82]. As a result, regardless of the notation used to collect and transmit data, measurements can be described using Semantic Web methods, creating a so-called Semantic Web of Things (SWoT) [83,84].

Proper semantization requires not only choosing the right markup, but also the right vocabulary. From both the Semantic Web and Data Mining perspectives, it is important to ensure that multiple datasets use the same set of vocabulary. In the case of weather data processing, this can allow for, for example, an unambiguous statement that both the *X* value from set $S_{1}$ and the *Y* value from set $S_{2}$ represent a measurement of air temperature 2 m above the ground. Appropriate metadata also allow us to determine that the *X* value is expressed in K and the Y value in ${}^{\circ }C$, which will facilitate their conversion to a common unit.

To address the latter issue, in [85], the authors introduce Custom Datatypes (CDT) [86]—a vocabulary based on The Unified Code for Units of Measure (UCUM; https://ucum.org/; accessed on 14 April 2021) for representing measurements along with their units in data semantized according to the RDF standard. It includes a general type of measurement (cdt:ucum) as well as more specific ones (e.g.,  cdt:temperature, cdt:pressure). The authors provide a working Java implementation that allows on-the-fly conversions performed during query execution to return results in the desired unit, regardless of the unit in which the measurement was stored (https://ci.mines-stetienne.fr/lindt/; accessed on 14 April 2021).

**Figure 1:**
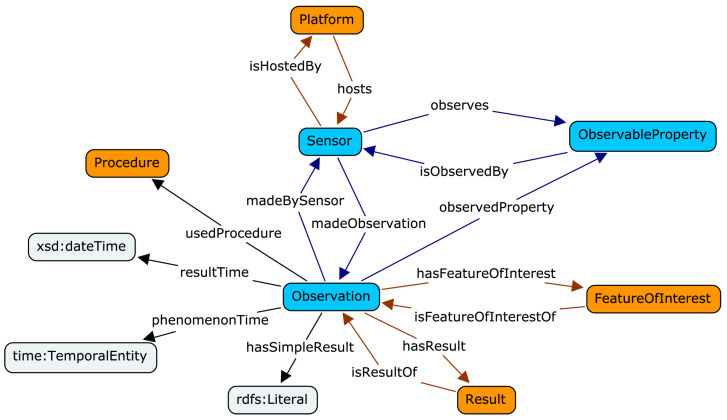
Observation and related concepts in SOSA ontology. Reprinted from [88], with permission from Elsevier.

To address the need for a standardized vocabulary for measurement representation, the W3C and OGC joined forces in the Spatial Data on the Web Working Group (for an overview of other approaches to sensory data semantization see [12]), which led to the development of the Semantic Sensor Network (SSN) ontology [87]. Its core concepts were further refined, leading to the creation of a lightweight self-contained Sensor, Observation, Sample and Actuator (SOSA) ontology [88]. From the ubiquitous sensing point of view, the key concept is the Observation. It has a Result of a Procedure performed at a specific time by a Sensor (e.g.,  thermometer) that observes some object (Observable Property, e.g., the air at the top of the Eiffel Tower) and measures a particular Feature of Interest (e.g.,  temperature), as summarized in Figure 1. The SSN and SOSA ontologies contain generic high-level terminology and thus can be further refined in more detailed application- and domain-specific ontologies, for example, expanded for the whole IoT area in an IoT-Lite ontology [89] or adapted to describe energy consumption related data [90].

The practical application and usefulness of data semantization in environmental data preprocessing may be illustrated using weather data. In this scenario, temperature, humidity, or rainfall data may come from multiple heterogeneous sources that need to be properly integrated [91,92]: private sensors connected via networks like Weather Underground (https://www.wunderground.com/; accessed on 14 April 2021), sensors managed by local authorities (e.g.,  [93]), data provided by (commercial) internet services (e.g.,  OpenWeatherMap) and data published as (Linked) Open Data.

In order to combine them into a single knowledge graph, it is necessary to first define an appropriate vocabulary and scheme. Then, a dedicated wrapper should be developed for each source to tag the data using the pre-defined scheme. In Listing 1, an example of a semantized data sample adapted from [94] is shown. It uses previously mentioned vocabularies: SOSA (sosa:) to represent observations and CDT (cdt:) to describe measured values. The custom namespace (weather:) is also used for other pieces of information. Finally, one can see the use of the GeoSPARQL (geo:) ontology [95]. The specification of the geographic coordinates according to this standard (see line 15 in Listing 1) makes it possible to easily search the dataset and find particular points, for example, those closest to a given location or all the points in a given area. Listing 2 shows a sample query that extracts all temperature data for the area of Kraków, PL. The filter in line 16 is responsible for selecting points from the indicated area (defined by a set of coordinates). In line 15, it is specified that the result should be given in Kelvin—automatic unit conversion will be done where necessary. Data generated in this way are therefore standardized and ready for further analysis. Depending on the needs, the semantic description can be more detailed, including, for example, sensor specifications and more metadata about the measurement site [94].

**Listing 1:**
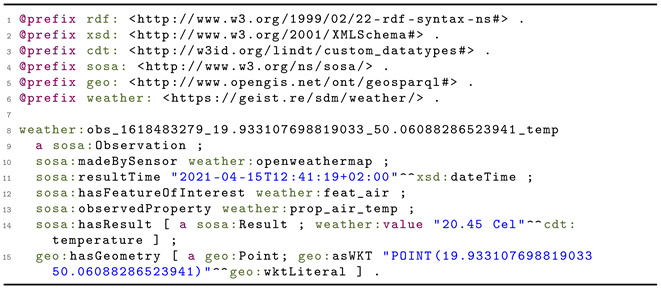
An example of a semantized data sample taken from OpenWeatherMap reporting air temperature at Jagiellonian University.

**Listing 2:**
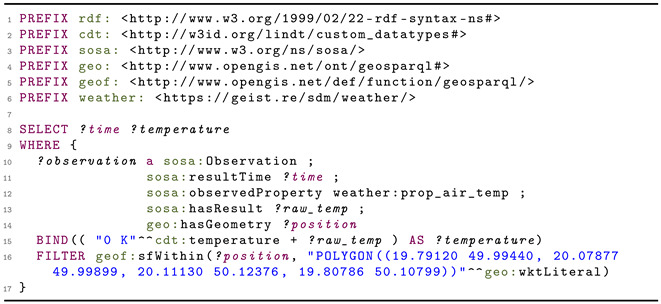
A sample query to extract all temperature measurements for the Kraków, PL area.

There are state-of-the-art frameworks that facilitate the entire process of sensory data semantization. The Linked Sensor Middleware (LSM) [96] provides both an automatic tagging mechanism by applying custom wrappers to data sources, and a web interface for manual annotation. The whole is complemented by a web service that allows for data extraction using the SPARQL query language. SWoT4CPS (Semantic Web of Things for Cyber-Physical Systems) [84], in turn, provides a more sophisticated ontology, which includes, for example, cause–effect relationships, that can be used not only for data semantization, but also for further processing and mining on a “semantic” layer of the system. For an overview of other sensing semantization frameworks, see [84].

Environmental sensing is not limited to the observation of changing weather conditions. Other applications include, but are not limited to, automatic temperature and humidity control with anomaly detection in smart buildings [84], early detection of collisions between pedestrians, cyclists and drivers to generate timely alerts on mobile devices [97], data collection from in-car sensors to predict generated noise, travel time and fuel consumption [98], and overseeing the food production process in the agri-food sector to reduce ${\mathrm{CO}}_{2}$ emission levels and energy consumption levels [99]. The applications discussed are summarised in Table 1.

An interesting area of environmental sensing is physiological sensing, which aims to measure various characteristics originating from the body. A diverse set of sensors, including smart bands, smartwatches [102], sensors embedded in phones [104], portable EEGs [103] and smart textiles [105,106], are used to measure temperature, heart action, respiration, galvanic-skin reaction, electroencephalography and other signals. Among the most common applications, there are stress levels and changes in emotions detection [100], assessment of involvement in education [101], health monitoring [102] and various cognitive enhancement tasks, including driver fatigue detection and the assessment of air traffic controllers’ mental fatigue [103].

### 3.2. Sensing in Industrial AI

**Figure 2:**
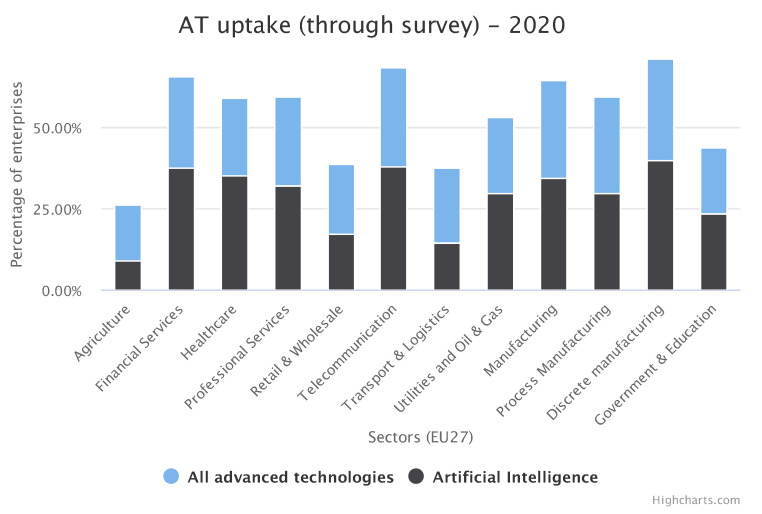
Percentage of advanced technology uptake in Industry 4.0 and AI in this uptake. Generated with https://ati.ec.europa.eu/data-dashboard (accessed on 8 April 2021).

**Figure 3:**
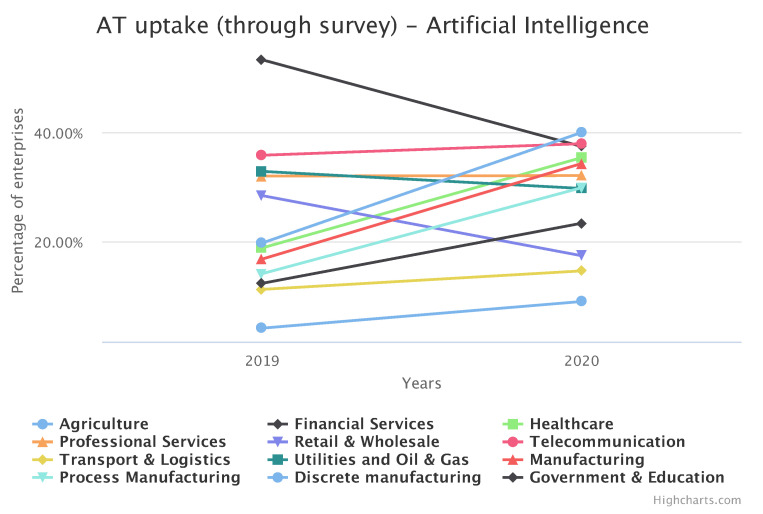
Trend in percentage uptake of advanced technology in 2019 and 2020. Generated with https://ati.ec.europa.eu/data-dashboard (accessed on 8 April 2021).

Environmental sensing is nowadays most extensively developed in the area of Industry 4.0 applications. Industry 4.0 (I4.0) defines an ongoing transformation of traditional business processes by the adaptation of new technologies and automation systems. Although the term originally referred only to manufacturing, currently it can be extended to almost every sector where technology plays an important role. Figure 2 depicts the advanced technology uptake in different sectors as of 2019. This can be considered as the expansion of I4.0 among enterprises, which is growing rapidly in almost all sectors, for example (discrete) manufacturing [107,108], especially in the context of the adaptation of Artificial intelligence as presented in Figure 3.

**Figure 4:**
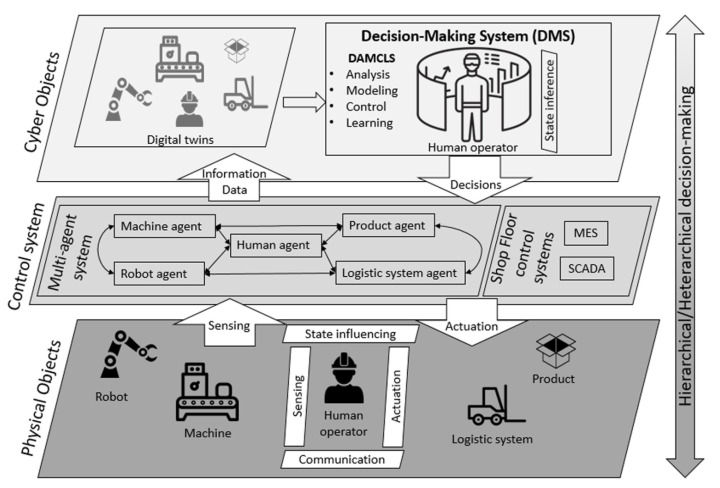
Three levels of the Cyber–Physical system in Industry 4.0 [110].

This allows us to extend the definition of I4.0, after [109], to a complex technological system that embraces a plethora of technologies, the implementation of which allows the development of intelligent manufacturing processes, composed by devices that are able to exchange information, perform actions and control each other. These technologies include, but are not limited to, Cyber–Physical Systems, Internet of Things, Robotics, Big Data, Artificial intelligence and so forth. The adaptation of such technologies in all of the cases is performed on many different levels of abstraction. Such levels can be generalised to three stages of process/data/control flow, as shown in Figure 4.

These three layers represent different levels of interaction of humans with the system, and hence different levels of knowledge and semantics’ exploitation by automated algorithms (including data mining and machine learning systems). At the Physical layer, humans directly interact with machines and other equipment. This is usually supported by built-in interfaces and does not require any additional layers to be fully operational. The control layer serves as a middleware between the Physical layer and the Cyber layer. It can be considered as a technical layer for exchanging and storing data from the machines and other system components, but also extends the control over the larger parts of the system, such as SCADA. The Cyber layer mirrors the physical environment that is formed by the concatenation of the Control system and Physical objects. This layer is mostly responsible for tasks related to the analysis of the data, modelling, learning, decision support, predictive maintenance and other high-level tasks [111].

Despite the automation of the system, humans still play an important role in each of the aforementioned levels and are mostly present in Physical and Cyber layers presented in Figure 4. Such a system, where a human operator is actively involved in the automated Cyber–Physical process is called a Human Cyber–Physical System [112]. In this work, we focus on the level where data acquisition, processing and utilization takes place; therefore, we reach in our discussion the moment where such systems are designed and built up to the deployment phase. Due to the broad nature of the problem, we narrowed the discussion to the areas in the process that are data-driven and, yet, the interaction with humans exists. This coexistence of the semantic world and the data driven world is especially difficult to embrace in the industrial process. Due to the fact that the knowledge that is possessed by human operators is rich in semantics but most often lacks formalism, the integration of such knowledge into the system is a challenging task.

**Figure 5:**
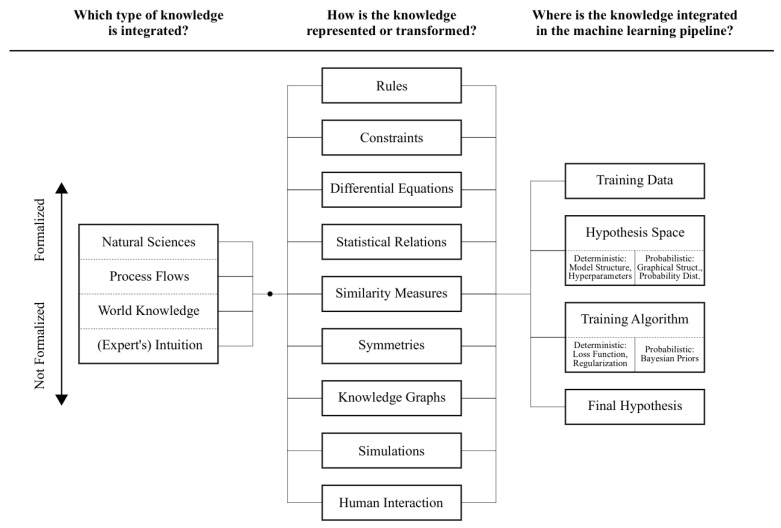
Knowledge source, its formalization and application to different ML/DM stages [8].

This coexistence is achieved with many different methods, depicted in Figure 5. In the following sections, a description of particular applications of selected methods in the area of I4.0 will be presented. They are also summarized in Table 2.

#### 3.2.1. Formalization of Semantics for Industrial AI Sensing

In Figure 5, different levels of formalization of knowledge are presented. Depending on the level of formalization, different methods for the knowledge transfer to machine learning pipeline can be chosen. In this section, we present how such knowledge can be used in practical applications. We will focus mostly on ontologies as one of the most expressive mechanisms for encoding and processing domain knowledge in many fields.

In [113], the integration of Semantic Web techniques in a large Industry 4.0 context was presented. The authors deployed the SANSA Stack to enable uniform access to Surface-Mount Technology (SMT) data. An ergonomic visual user interface was proposed to help non-technical users coping with the various concepts underlying the process and conveniently interacting with the data. In [114], the authors propose a framework for constructing a semantically annotated knowledge graph for Industry 4.0 related standards called I40KG. The graph provides a Linked Data-conform collection of annotated, classified reference guidelines, supporting newcomers and experts alike in understanding how to implement Industry 4.0 systems. The authors illustrate the suitability of the graph for various use cases, its already existing applications, and present the maintenance process and evaluate its quality. In [115], the authors provide a practical example and evaluation of the Bosh implementation of I40KG. In [116], the authors present a Semantic Web of Things for Industry 4.0 (SWeTI) platform, which is a cross domain, cross platform solution. In their approach, they extend the hierarchy of I4.0 layers by adding on top of the cyber layer the data analytic and application layers, which are supposed to be the main components for knowledge exchange. It serves as an intelligent interface with all of the knowledge hidden within the bottom layers. As this is only a framework, it does not provide any specific ontology but allows integration with domain knowledge formalized in such a way at the level of the cyber layer.

The remaining components for knowledge transformation and encoding are presented in Figure 5 and do not require complex frameworks such as knowledge graphs; however, they may lack the expressive power of the former in many cases. In the following section, we discuss the application of selected knowledge embedding methods to different phases in the ML/DM pipeline.

#### 3.2.2. Knowledge Embedding Methods

Knowledge embedding within the ML/DM pipeline can be conducted at many different stages, as depicted in Figure 5. Depending on the stage of the ML/DM workflow, different methods are more applicable. This natural consequence yields serious difficulties in communication and knowledge transfer between ML/DM phases. Below, we discuss methods that allow the integration of specific knowledge into the ML/DM pipeline.

Simulations, equations, statistical relations similarity measures or symmetries are mostly used in the first two phases of the DM/ML process. They usually do not use any formal knowledge representation that is utilised by data mining algorithms, as they are implemented within the pipeline or algorithm by data analysts and experts. In [117], the authors present a differentiable physics engine that can be integrated as a module into a deep neural network as a layer in its architecture to improve overall performance. Similar exploitation of physics equations is presented in [118], where the authors embed Gaussian process regression with stochastic equations that model the well-defined physics of the power grid dynamics. In [120], the authors approach the problem from a different perspective and embed domain knowledge that can be generated with simulations within a surrogate neural network that produces the approximation of simulations, which can later be used at further stages of the ML/DM pipeline.

In [121], the authors present a framework for ML/DM tasks configuration, which is based on the profiling that is generated through interactions with users. The knowledge representation and inference was not specified, while more emphasis was placed on the aspect of knowledge mediation involving the user/data scientist. They extended their work in [122] by showing how the profiles and user needs and expectations can be modelled and used in the ML/DM pipeline, for example, in a form of constraints. The same knowledge representation technique was presented in [123], where constraints were applied to the output space of classification neural network architecture to minimize the need to label the data, which might be costly. In [119], the authors approached a similar problem of using machine learning models and statistical learning on datasets that are relatively small. They demonstrate how embedding domain knowledge for the machine learning of complex material systems can improve its performance in the case of small datasets.

A comprehensive review on usage knowledge graphs in Industry 4.0 was presented in [136]. In [124,125], the authors present an approach for knowledge fusion in manufacturing operations with the use of knowledge graph embedding methods. Such knowledge can later be used by an arbitrary ML model for further training and decision making. A similar approach was presented in [126], where the system for predicting the geographic centers of fuel cells is enhanced with knowledge gathered form heterogeneous sources and unified in a form of RDF-based knowledge graph. The knowledge is used to generate training data for machine learning models that implement predictive maintenance tasks.

Human interactions are considered informal knowledge transfer methods that can be used to infer new knowledge by an algorithm, or to translate them into more formalized queries that can be processed by a machine. Such systems are usually built on top of one of the aforementioned formalisms. A comprehensive review of methods that allow the combination of virtual reality and augmented reality visualization techniques with machine learning and knowledge graphs was provided in [137].

#### 3.2.3. Decision Explanation Methods

An emerging field of science in recent years is eXplainable Artificial Intelligence (XAI). As stated in Section 2.3, one of the most important roles of these methods is to reverse the process of knowledge transfer. While the majority of the methods described in the previous section focus on incorporating domain knowledge into the ML/DM tasks, the goal of XAI methods is to translate the decision making process performed by an ML algorithm in a way that can be understood by the data scientist or domain expert. This can either be accomplished by augmenting and/or contextualizing the provided decision with additional information, such as background knowledge or similar/historic applied cases, or with domain knowledge providing an extended context, for example including declarative knowledge  [138]. Ultimately, this bridges the gap between knowledge discovery and data mining to decision support, and its contextualization, ultimately enabling computational sensemaking, for example, see [138].

In many cases, this requires the embedding of the raw decision with additional semantics that will be aligned with the expectations of the addressee. In many task-specific solutions, such explanations are delivered partially with the use of formalism for the ML model, for example, knowledge graphs. In [127], the utilisation of the knowledge graph was expanded. It not only serves as an input for the ML model, but can also be extended by statistical learning methods, enriching knowledge about the domain or ML decisions. Similar approaches for application in the Cyber–Physical system were also provided in [128,129,130]. In [131,132], the authors provide a method that aims to bring more semantics to clusters discovered by automated methods in an Industry 4.0 setting. Such semantic information can then be represented, for example in the form of rules, and can be used to extend the knowledge about the machinery states in the Cyber–Physical setting. In addition, [139] presented a method for visualizing interesting parts of the decision space of a model in order to make the respective modeling and ultimate decisions interpretable for humans. Further directions include the application of interpretable methods for obtaining explanatory patterns—for contextualization and explanation, for example, see [133,134,135].

### 3.3. Social Sensing

Social sensing [140,141,142,143] relates to observing human interactions and capturing the respective individual and/or collective behaviors by way of sensors, which can, for example, relate to both offline as well as online sensors, for example, [144,145,146,147,148,149,150,151] In the following, we first outline the general area of social sensing, before discussing two specific subareas: First, we consider *semantic social sensing* relating to the use of strongly formalized knowledge structures for integrating semantic information, such as ontologies, into the respective mining and analysis approaches; second, we discuss *semantic social network analysis*, focusing on the analysis methodology of social network analysis applied to rich social sensing data. Here, we also revisit semantics given light-weight knowledge structures, that is, collaborative tagging leading to folksonomies for integrating (semantic) information. Table 3 summarizes the discussed semantic social sensing and social network-based frameworks/platforms.

#### 3.3.1. Social Sensing in Ubiquitous and Social Environments

For social sensing in ubiquitous and social environments, a variety of heterogeneous sensor data can be observed and analyzed, for example, considering specific sensors, social media and the ubiquitous social web, and so forth, cf.  [147,161,162,163]. This specifically relates to observing human interactions, that is, social and physical activities [140,144,145,148,151,164]. While [161,162], for example, describe social sensing on the ubiquitous social web, [165] discusses social sensing in the context of social media and human face-to-face interactions, using the OpenBeacon badges of the SocioPatterns consortium [144]. Other prominent sensors for social sensing in similar contexts include the Sociometric badges [145] and successors such as the Rhythm badge [164]. Besides data mining on social interaction networks [166], social sensing is also relevant for (computational) social science and digital epidemiology [149,167], as well as for applications in human sensing in industrial contexts, such as manufacturing [168].

Regarding the issues of data modeling, mining and analysis in social sensing, we follow the presentation in [166] and focus on *social interaction networks* [169,170,171,172]. These enable a wide range of modeling and analysis options, cf. [166,171,173], that is, user-related social networks capturing social relations inherent in social interactions, social activities and other social phenomena which either directly connect users or act as proxies for social user relatedness. This then also includes interaction data from sensors and mobile devices, as long as the data are created by real users. In this way, social sensing transcends offline scenarios using hardware sensors (only) such as in mobile and ubiquitous computing, and can be linked to virtual sensor data as well—that is, data captured in online contexts. For social sensing in such broadly defined contexts, we can thus consider, for example, users who connect their mobile phones via Bluetooth, interact similarly with online applications such as Flickr, communicate in a similar way (or about similar topics) on Twitter or Facebook, or explicitly establish “contacts” within certain social applications, for example, [173,174]. Furthermore, we consider real-world contacts as determined by other ubiquitous computing applications [175,176], the ubiquitous web [161,177,178], and the principle of object-centric sociality [179], where objects of a specific actor—for example, resources—mediate connections to other actors.

#### 3.3.2. Semantic Social Sensing

Depending on the respective types of social sensing data, in particular when different representations are integrated in a multi-modal strategy, different representations can be derived. This is possible, for example, when including sequential/time series sensor data together with unstructured information from web pages and structured information from ontologies. For example, [180] proposes such an approach called *semantic social sensing*, making use of ontologies and semantic augmentation together with textual analysis on user generated information such as comments. Such data representations are necessary in order to prepare the analysis, drive explanations, or guide exploratory approaches. Tabular (structured) data can usually be mapped and normalized in a straight-forward way, whereas unstructured (e.g.,  text) and semi-structured data need further processing. Here, information first needs to be extracted or abstracted. In this respect, including background and/or domain knowledge in such approaches becomes more and more important; to improve the models, to drive explanations and, ultimately, to allow *computational sensemaking*.

Regarding formalized knowledge, providing this in the context of sensing with respect to smart cities—ontological requirements and useful semantic information as an ontological representation of urban data—has been discussed in [152,153]. Furthermore, [154] presents the modeling of smart sensors on top of the SOSA/SSN ontologies [87,88] (as already introduced above), also with the semantic smart sensor network (S3N) modular ontology. In [181], the authors present semantic social sensing applications in the form of a semantic sensing middleware for the Web of Things. In [155], the authors introduce an ontology for hybrid semantic sensor networks (HSSN) which extends the Semantic Sensor Network (SSN) ontology described above for, for example, more heterogeneous sensors and platforms in order to enable extended analysis and mining. Furthermore, [156,157,182] specifically discuss semantic social network analysis for modeling social interaction networks in order to create richer (semantic) models given the social sensing data, which can also be used for detailed analysis. Basically, a network between humans, ontologies and their interlinks is provided and analyzed, also taking semantic information on the respective graph structure into account.

Applications of such approaches include the analysis of organizational social networks [159] or data mining for recommender systems, for example, see [183]. Specifically, in [184], a data mining method for generating recommendations in the context of software development is provided. Given social sensing data, as well as additional domain knowledge and information extracted from CVS logs, the performance of the recommendations could be significantly improved, combining both sources of information and knowledge.

Altogether, using semantic social sensing, for example, unusual social activities can be detected using geo-tagged microblogs [185], or human activity and interesting patterns can be analyzed using ubiquitous social data in social [186,187] or urban contexts  [188,189]. Here, as mentioned in [142], it is particularly important to include rich information, such as place and space semantics, about the respective social and spatial interactions. Semantic signatures/labels can also be applied [189]; example applications include the health care domain considering the detection/monitoring activities of daily living (ADL) [160,190,191,192]. Here, rule-based approaches are also relevant [193,194].

#### 3.3.3. Semantic Social Network Analysis

Overall, the analysis of online social network data has received significant attention for analyzing large and complex systems, such as large-scale social network systems or the internet infrastructure, and so forth cf.  [195,196,197]. While there has been foundational work on social network analysis and mining on social sensing data, such as the analysis of face-to-face contact networks, for example in [144], semantic data mining on those networks is still a rather new field of research. When integrating semantic information into social network analysis approaches, this leads to *semantic social network analysis* [198].

Regarding the semantic information to be included in data mining, we distinguish different types, for example, formalized in ontologies, taxonomies, or folksonomies. Semantic structures emerge from collaborative tagging, which can be used at the level of the respective tagged objects, such as images or locations on a map. These structures are called folksonsomies [199,200]. In the scope of social sensing, a folksonomy is also called a *sensonomy* [201,202], for example, for urban sensing. Then, we can apply this on the level of maps and data mining to the interactions, spatial structures and so forth. An example is the analysis of social interaction networks integrating multi-modal sensing information, for example from the WideNoise and Airprobe systems, implemented using the Ubicon system [151,203], for observing and analyzing social and physical activities. Here, semantics are applied in the form of subjective information, in addition to folksonomies, as collaborative tag vocabularies.

## 4. Summary, Challenges and Future Directions

Today’s sensing technology often utilizes diverse hardware sensors that generate huge volumes of heterogeneous data—Big Data. Therefore, the processing of these data is mostly performed by the use of data mining methods and tools. However, proper interpretation of these data often requires the inclusion of certain knowledge regarding the operational context of data acquisition or requirements of a specific domain in which the sensory system is used. The objective of this survey was to provide a concise overview of a range of approaches that aim to extend the typical data mining process with the use of semantic information, the introduction of knowledge and declarative representations. We refer to these approaches using the general term *semantic data mining*. We also emphasized the role of interpretability and explainability in data mining, which can be achieved with the use of semantic data mining.

Furthermore, we discussed selected applications of data mining for ubiquitous sensing, where—in our opinion—semantic interpretation of data can be particularly useful. We selected three specific areas of interest for which we described a number of approaches categorized based on the sensing framework used, semantic formalism, explainability and domain. The first area is environmental sensing, for which we mentioned standardized protocols and discussed their use for weather data and physiological sensing. The second important area is industrial AI, for which we considered how different knowledge sources can be formalized and applied for data mining. The third area is social sensing, including semantic social sensing, and semantic social network analysis.

It is worth noting that the adoption of the semantic data mining approaches we discussed may face different *challenges*, which we identified—from the surveyed papers, as well as from our discussion. Thereby, we deduce the following challenges, which we present in order to inspire future research in these areas:

1. The first challenge is related to the availability of domain knowledge, its form and representation. Semantic data mining approaches differ with respect to the knowledge representation used, for example, from simple annotations to formalized knowledge models. This selection also has an impact on the possible cognitive load of human experts participating in the knowledge acquisition process. Furthermore, in certain domains, formalized knowledge is in fact present in the form of rules, constraints, structures and vocabularies. The introduction of such knowledge into the DM process—if successful—can allow for the alignment of the results of the process with the domain requirements.
2. The second important challenge is the proper selection of the phase of the DM where the knowledge is introduced. As we discussed, it is often the case that preliminary stages of the process are very time consuming, so a proper understanding of the data can be achieved. This is why the use of domain knowledge in this stage could be beneficial, for example, as a part of the feature engineering activity. However, in practice, such an approach—while possible—is often overlooked.
3. The third challenge is related to the provision of explainability methods. The use of complex black-box machine learning models that offer superior accuracy can result in certain risks in terms of their interpretability. The need to formulate explanations instrumental for understanding the results of the DM process and for putting it in the context of specific domains, is an important requirement. As such, the use of semantic data mining methods can be of particular interest and value for interpretability and explainability, as we have also discussed throughout the methods and application sections.

To summarize, we expect in the near future a growing interest in semantic data mining approaches, especially in ubiquitous sensing. Specific future directions of the introduction of semantic methods in data mining could include, for example: semantic interoperability in sensing for limiting data pre-processing; semi-automated methods for data annotation in the early phases of the data mining process; domain knowledge modelling during data acquisition (possibly as part of the feature engineering); emphasis on explanations regarding both the data and the output of the data mining process, and so forth.

In addition, since both symbolic and sub-symbolic data mining and machine learning methods can be applied in the context of ubiquitous sensing, domain knowledge—enabling semantic data mining—can be added as a third dimension in order to allow for a fruitful combination of those different methods, and also potentially to serve as a strong promoter of their combination and application in sensing scenarios. With the above mentioned observations in mind, we envisage the increasing adoption of semantic data mining in research as well as in the wide range of fields of application.

## Figures and Tables

**Table 1:** Semantic Web of Things (SWoT) for Environmental Sensing [10,12,83,84].

Ref.	Sensing Framework	Semantic Formalism	Explainability	Domain
[91]	Custom	Simple taxonomy	Visual interface	Smart City
[92]	Custom	Ontology	No	Smart City
[94]	Custom	Ontology (SOSA, SSN)	Visual interface	Smart City
[97]	Custom	Relational database	Visual interface	Traffic
[98]	Custom	Ontology (O&M)	Visual interface	Traffic
[99]	None	*Out of the scope*	Visual interface	Agri-food
[100]	None	*Out of the scope*	No	Emotions
[101]	None	*Out of the scope*	No	Education
[102]	Custom	Relational database	Visual interface	e-Health
[103]	None	*Out of the scope*	No	Fatigue detection
[96]	LSM	Ontology (SSN)	Visual interface	cross-domain
[84]	SWoT4CPS	Ontology (SSN), rules	No	cross-domain

**Table 2:** Sensing platforms in Industrial AI.

Reference	Sensing Framework	Semantic Formalism	Explainability	Domain
[113]	SANSA stack	Semantic Web	Visual interface	domain-specific (electronic mounting)
[114,115]	I40KG framework	Ontologies	No	cross-domain
[116]	SWeTI framework	Semantic Web	No	cross-domain
[117,118,119]	None	Physics equations	No	domain-specific
[120]	None	Physics approximation model	No	domain-specific
[121,122,123]	Custom	Constraints	visualization dashboard, knowledge mediation	cross-domain
[124,125,126]	Custom	knowledge graph	No	cross-domain
[127,128,129,130]	None	knowledge graph	knowledge-graph extensions	cross-domain
[131,132]	None	Rules	Shapely values	domain-specific
[133,134,135]	None	knowledge graph	visual, symbolic, statistical	cross-domain

**Table 3:** Semantic Social Sensing and Social Network-Based Frameworks/Platforms.

Ref.	Sensing Framework	Semantic Formalism	Domain/Sensing
[91]	Custom	Ontology	Smart City
[152,153]	Custom	Ontology	Smart City
[154]	Custom	Ontology (SOSA, SSN)	IoT/Heterogeneous Sensors
[155]	Custom	Ontology	IoT/Heterogeneous Sensors
[156,157]	None	Ontology	Social Networks
[150]	Custom	Folksonomy-Based	Social Network/Human Sensors
[151]	Custom	Folksonomy-Based	Social/Human Sensors/IoT
[144,158]	Custom	Folksonomy-Based	Social/Human Sensors
[159]	None	Network-Based	Social/Textual/User-Generated Content
[160]	Custom	Ontology	Healthcare
